# 한국 응급실 간호사의 전문직 자아개념, 팀워크가 직무만족에 미치는 영향: 횡단적 조사연구

**DOI:** 10.7586/jkbns.25.015

**Published:** 2025-05-22

**Authors:** Jeong Yeon Hwang, Sunjoo Boo, Sun Hyoung Bae, Eun Ji Seo

**Affiliations:** 1 Administrative Education Team, Yongin Severance Hospital, Yongin, Korea; 1 용인세브란스병원 행정교육팀; 2 College of Nursing · Research Institute of Nursing Science, Ajou University, Suwon, Korea; 2 아주대학교 간호대학 · 간호과학연구소

**Keywords:** Self concept, Crew resource management, healthcare, Job satisfaction, Emergency responders, 전문직 자아개념, 팀워크, 직무만족, 응급실 간호사

## Abstract

**Purpose:**

This study aimed to derive insights into professional self-concept, teamwork, and job satisfaction among emergency department (ED) nurses and to prepare basic data for improving job satisfaction among ED nurses.

**Methods:**

The study was conducted between October 2023 and April 2024 at two certified tertiary hospitals and one general hospital in Gyeonggi-do, Korea, among 124 ED nurses. Data were analyzed using SPSS version 26.0, and the analysis included descriptive statistics, the t-test, Pearson correlation, and multiple regression.

**Results:**

The factors affecting job satisfaction in ED nurses were professional self-concept (β = .66, *p* < .001), teamwork (β = .17, *p* = .003), and ED work satisfaction (β = .17, *p* = .003), and the overall explanatory power of the model was 67.7%.

**Conclusion:**

This study makes a significant contribution by demonstrating that professional self-concept and teamwork can increase job satisfaction in ED nurses. Therefore, as a strategy to increase job satisfaction through improving ED nurses’ professional self-concept and teamwork, effort should be made to apply team simulation programs that provide education and self-improvement opportunities for frequent diseases and situations encountered in the ED, and to communicate effectively about patient treatment strategies.

## 서론

### 1. 연구의 필요성

응급실은 생명이 위급한 환자들이 내원하는 상황에서 간호사의 즉각적이고 집중적인 간호와 신속한 업무처리가 요구되는 곳이다[1]. 응급의료 통계연보에 따르면 2023년 인구 천 명당 전국 응급실 이용 건수는 187.9건으로 2022년 대비 15.4건 증가하여 응급실 이용자는 늘고 있는 추세이다[2]. 권역응급의료센터의 지정과 응급의료기관 평가 등을 통해 응급실 인력이 보강되고 응급실 근무환경과 업무 절차가 체계화되고 있으나[3], 시간을 다투는 응급실의 업무 특성으로 인해 의료진들은 항상 긴장된 상황에서 근무하게 된다[4].

즉각적인 간호가 필요한 응급환자가 불규칙적으로 내원하는 응급실 상황은 응급실 간호사의 직무만족에 부정적인 영향을 미칠 수 있다[5]. 응급상태의 환자들로 인해 간호업무량과 간호 수행에 대한 부담이 크고 환자 및 보호자, 타부서 의료진과의 복잡한 대인관계로 인해 직무수행 시 부정적인 상황을 경험할 수 있기 때문이다. 때문에 응급실 간호사의 높은 직무만족은 업무수행과 근로의욕을 증진시키고 개인의 사기 향상과 병원 조직의 목표달성에 영향을 미치며 환자에게는 양질의 간호를 제공할 수 있으므로 타부서에 비해 그 중요도가 더 높다[5]. 즉, 응급실 간호사의 직무만족이 높으면 직무에 대한 태도가 향상되어 자신의 업무에 대해 더 잘 이해하게 되어[6], 환자의 안전과 간호의 질 향상에도 영향을 줄 수 있다[4]. 그러나 응급실 간호사의 직무만족 수준은 타부서에 비해 낮은 것으로 보고되고 있다[5,7].

전문직 자아개념은 전문직으로서 자신이 갖는 스스로에 대한 정신적 지각으로 자신이 수행하는 직무에 대해 느끼는 견해를 말한다[8]. 간호사의 전문직 자아개념이 높을수록 직무만족이 향상될 수 있는데, 간호 업무에 대한 자신감과 역량이 높아지고 교육에 대한 욕구가 증가하여 환자 치료에 긍정적인 영향이기 때문이다[7]. 특히, 응급실 간호사는 병태생리적 지식 및 전문지식에 따른 체계적인 분석을 바탕으로 응급환자를 분류하고 우선순위를 신속하게 결정해야 하므로, 전문직 자아개념이 높으면 특수한 상황에서 어떠한 행위를 수행할 수 있다는 개인의 믿음과 자신감이 높아져 직무만족도가 향상된다[9]. 전문직 자아개념이 높은 간호사는 간호 업무에 대해 더 많은 책임감과 기초간호에 대한 지식을 바탕으로 환자를 더 관심 있게 보게 되어 환자 안전에도 긍정적인 영향을 미치며, 직무에 대한 만족이 높아져 간호직을 유지하려는 경향을 보인다[10]. 따라서 응급실 간호사가 긍정적인 전문직 자아개념을 확고히 가질 때 자신의 전문적 업무를 더 잘 수행할 수 있어[11] 직무만족을 높이는 요인이 될 수 있다.

응급실은 타부서에 비해 빠르고 정확한 의사결정이 요구되는 만큼 응급실 의료진 간 팀워크는 응급실 간호사의 직무만족에 중요한 부분이다. 환자 치료라는 공동의 목표와 관련된 공동체 의식은 팀으로서 보다 생산적이고 효율적으로 일할 수 있는 역량과 책임감을 강화하며[12], 긍정적인 팀워크 인식은 공동의 목표 공유와 달성을 위한 공동 작업에 적극적으로 참여할 수 있게 한다[13]. 간호사가 인식하는 팀워크 정도가 높을수록 동료들과 최대한 갈등 없는 관계를 구축하여 높은 직무만족을 가지고 업무를 수행할 수 있다[14]. 또한 효율적인 팀워크는 치료 계획을 더 잘 이해할 수 있어 개인의 역량 발휘에도 긍정적인 영향을 미쳐 간호사의 직무만족을 높일 수 있는 요인이다[15]. 응급실 간호사의 팀워크 인식이 높을 때 긍정적인 태도로 업무에 임하며 기꺼이 돕고자 하는 태도를 통해 근무를 더 즐겁게 수행할 수 있도록 도와주므로[16], 팀워크 또한 직무만족의 긍정적 영향요인이 될 수 있다.

선행연구에서 직무만족에 영향을 미치는 요인으로 임상간호사를 대상으로 전문직 자아개념과 직무만족도에 대한 연구[9,17], 팀워크와 직무만족도에 대한 연구[17,18] 등이 진행되었으나, 응급실 간호사를 대상으로 전문직 자아개념과 팀워크가 직무만족에 미치는 영향을 파악한 연구는 부족한 실정이다. 응급실 업무의 특성 상 간호사로서 전문성과 효과적인 팀워크는 응급환자에 대한 대처를 효과적으로 빠르게 할 수 있으므로 간호사의 직무만족이 향상될 수 있다. 이에 본 연구는 응급실 간호사의 직무만족에 전문직 자아개념과 팀워크의 영향을 파악하여 응급실 간호사의 직무만족 향상을 위한 기초자료를 제공하고자 한다.

### 2. 연구의 목적

응급실에서 근무하는 간호사의 직무만족에 영향을 미치는 영향요인을 파악하기 위함이며, 구체적인 목적은 다음과 같다.

(1) 응급실 간호사의 일반적인 특성에 따른 직무만족의 차이를 파악한다.

(2) 응급실 간호사의 전문직 자아개념, 팀워크와 직무만족 정도를 파악한다.

(3) 응급실 간호사의 전문직 자아개념, 팀워크와 직무만족 간의 관계를 파악한다.

(4) 응급실 간호사의 직무만족에 미치는 영향요인을 파악한다.

## 연구 방법

### 1. 연구 설계

응급실 간호사를 대상으로 전문직 자아개념과 팀워크가 직무만족에 미치는 영향을 규명하기 위한 횡단적 조사 연구이다.

### 2. 연구 대상

본 연구의 대상은 응급실 간호사로, 총 임상 경력이 6개월 미만인 경우, 간호단위 관리 업무를 맡고 있는 수간호사 이상의 관리자는 연구 대상자에서 제외하였다. 본 연구에 필요한 표본의 크기는 G*Power program 3.1.9.7 [19]을 이용하여 회귀분석에서 사용되는 통계적 검정법에 따라 유의수준 0.05, 검정력 0.80, 중간 효과 크기 0.15, 예측변수 9개일 때 필요한 대상자 수는 최소 114명이었다. 탈락률 10%를 고려하여 127부를 모집하였고, 불충분한 응답 설문지 3부를 제외하고 124부를 최종 자료 분석에 이용하였다.

### 3. 연구 도구

#### 1) 전문직 자아개념

전문직 자아개념은 Arthur [20]가 개발한 전문직 자아개념 측정도구 Professional Self Concept of Nurses Instrument를 Sohng과 Noh [21]가 번역한 측정 도구로서, 전문적 실무(융통성, 지도력, 기술), 만족감, 의사소통을 포함한 3가지 하위 영역의 총 27문항으로 구성되었다. ‘그렇지 않다’ 1점에서 ‘그렇다’ 4점까지 Likert 척도로 측정되며 평점평균 점수가 높을수록 전문직 간호사로서 자신의 업무에 대한 스스로의 느낌과 견해가 긍정적임을 의미한다. 도구의 신뢰도는 개발 당시 Cronbach’s alpha 값은 .85이었고[20], 국내 간호사를 대상으로 연구한 Sohng과 Noh [21]의 연구에서 .85, 본 연구에서는 .84이었다.

#### 2) 팀워크

팀워크는 Larson과 Lafasto [22]가 개발하고 Kim [23]이 한국어로 번역하고 재구성한 척도를 Cho [24]가 간호사의 상황을 반영하여 문맥을 수정한 측정 도구로서, 목표공유, 결과지향, 상호협력을 포함한 3개의 하위영역의 총 24개 문항으로 구성되었다. ‘전혀 그렇지 않다’ 1점에서 ‘매우 그렇다’ 5점까지 Likert 척도로 측정되며 평점평균 점수가 높을수록 지각된 팀워크가 좋음을 의미한다. 도구의 신뢰도는 개발 당시 전체 문항의 신뢰도는 확인하지 않았고 하부영역의 신뢰도는 목표공유 Cronbach’s alpha .87, 결과지향 Cronbach’s alpha .89, 상호협력 Cronbach’s alpha .93이었다[22]. 간호사를 대상으로 연구한 Cho [24]의 연구에서 전체 문항 신뢰도는 Cronbach’s alpha 값은 .96이었고, 본 연구에서는 .96이었다.

#### 3) 직무만족

직무만족은 Lee 등[25]이 국내 임상간호사를 대상으로 개발한 직무만족 측정 도구로서, 조직적 차원의 지원과 인정 9문항, 직업을 통한 인격적 성숙 6문항, 존중 및 인정의 인간관계 8문항, 전문직 간호사로서 책무완수 4문항, 직업의 안정성과 보람 3문항, 전문직 역량 발휘 3문항의 총 33문항으로 구성되어 있다. ‘전혀 그렇지 않다’ 1점에서 ‘매우 그렇다’ 5점까지 Likert 척도로 측정되며 평점평균 점수가 높을수록 직무만족이 긍정적임을 의미한다. 도구의 신뢰도는 개발 당시 Cronbach’s alpha 값은 .95이었고[25], 본 연구에서는 .95이었다.

### 4. 자료 수집

경기도 소재 대학병원(아주대학교병원, 성빈센트병원, 용인세브란스병원) 응급실 간호사를 대상으로 2023년 10월 10일부터 2024년 4월 23일까지 자료수집을 시행하였다. 자료수집을 위해 연구자가 해당 기관의 간호부에 연구의 목적과 절차에 대해 설명하고 협조를 구한 후 진행하였다. 각 병원 응급실 내 게시판에 응급의료센터에 근무하고 있는 응급실 간호사를 대상으로 작성된 모집안내문을 게시하였고, 모집안내문의 QR code에 링크된 구조화된 온라인 자가 보고 설문지를 사용하여 자료를 수집하였다. 설문 작성 전 설명문에 연구의 목적과 내용, 연구 절차 및 방법, 연구 참여에 따른 이득이나 중단 가능성 및 연구 대상자의 사생활과 기밀 유지에 관한 사항을 안내하였고, 수집된 자료는 오직 연구 목적으로만 사용된다는 내용을 제공하였다. 본 연구에 참여하는 것으로 자발적으로 동의 버튼을 누르면 설문이 시작되도록 하였고 모든 응답을 마친 연구참여자가 설문지 제출을 누르면 최종 참여가 완료되도록 하였다.

### 5. 자료 분석

수집된 자료는 SPSS version 26.0 (IBM Corp., Armonk, NY, USA) 통계프로그램을 사용하였으며 구체적인 방법은 다음과 같다

• 연구대상자의 일반적 특성, 전문직 자아개념, 팀워크, 직무만족은 기술통계로 분석하였다.

• 연구대상자의 일반적 특성에 따른 직무만족의 차이는 independent t-test, one-way ANOVA로 분석하였다.

• 연구대상자의 전문직 자아개념, 팀워크와 직무만족 간의 관계는 상관관계분석을 하였다.

• 연구대상자의 직무만족에 미치는 영향요인을 파악하기 위해 다중회귀분석을 이용하였다.

### 6. 윤리적 고려

연구수행에 앞서 본 연구에 참여하는 대상자를 보호하기 위해 각 병원의 기관 윤리 심의 위원회의 승인(IRB No: SB-2023-481; 9-2023-0067; 신20240404-003)을 받은 후 자료수집을 하였다. QR code로 연결되는 구조화된 온라인 자가 보고식 설문지를 이용하여, 연구 참여 설명서와 동의서가 기재된 화면을 읽고 연구 참여에 대한 동의 버튼을 누르는 경우 설문으로 들어가도록 설정하였다. 설문 참여 도중 그만 두고 싶을 경우 창을 종료하여 언제나 중단 가능하며, 제출을 클릭하기 전에 응답한 내용은 자동으로 영구삭제되었다.

## 연구 결과

### 1. 대상자의 일반적 특성에 따른 직무만족의 차이

본 연구대상자의 일반적 특성과 이에 따른 직무만족의 차이는 Table 1과 같다. 총 124명 중 여자가 87.1%로 남자보다 많았고, 평균 연령은 29.79세이며, 대상자의 76.6%가 미혼이었다. 최종 학력은 4년제 대학 졸업(83.9%)이 대부분이었다. 권역응급의료센터에서 근무하는 응급실 간호사가 61.3%이었고 직위가 일반간호사인 대상자가 76.6%로 많았다. 총 임상 경력은 평균 6.24년으로 5년 이상에서 10년 미만인 대상자(36.3%)가 가장 많았고, 응급실 근무경력은 평균 4.95년으로 5년 이상에서 10년 미만인 대상자가 35.5%로 가장 많았다. 응급실 근무 만족도는 ‘만족한다’가 62.9%로 가장 많았다.

직무만족은 응급실 근무 만족도(t = 5.62, *p* < .001)에서 통계적으로 유의한 차이가 있는 것으로 나타났다. 즉, 응급실 근무 만족도가 만족인 경우 불만족인 대상자보다 직무만족이 높게 나타났다.

### 2. 대상자의 전문직 자아개념, 팀워크, 직무만족 정도

본 연구대상자의 전문직 자아개념, 팀워크, 직무만족 정도를 분석한 결과는 Table 2와 같다. 대상자의 전문직 자아개념은 4점 중 평점 2.63 ± 0.33점으로, 각 하위 영역별로는 전문적 실무, 의사소통, 만족감 순이었다. 대상자의 팀워크는 5점 중 평점 3.45 ± 0.70점으로, 각 하위 영역별로는 목표공유, 결과지향, 상호협력 순이었다. 직무만족은 5점 중 평점 3.46 ± 0.59점으로, 각 하위 영역별로는 전문직 간호사로서 책무완수와 전문직 역량 발휘가 가장 높았고, 직업의 안정성과 보람, 직업을 통한 인격적 성숙, 존중 및 인정의 인간관계, 조직적 차원의 지원과 인정 순이었다.

### 3. 전문직 자아개념, 팀워크와 직무만족의 상관관계

본 연구대상자의 전문직 자아개념, 팀워크, 직무만족의 상관관계는 Table 3과 같다. 대상자의 직무만족은 전문직 자아개념(r = .80, *p* < .001), 팀워크(r = .50, *p* < .001)와 양의 상관관계가 있는 것으로 나타났다.

### 4. 직무만족 영향요인

본 연구대상자의 직무만족에 영향을 미치는 요인을 파악하기 위해 일반적 특성 중 직무만족에 유의한 차이를 보인 응급실 근무 만족도를 더미변수로 변환하고, 상관관계를 보인 전문직 자아개념과 팀워크가 직무만족에 미치는 영향을 보기 위하여 다중회귀 분석한 결과는 Table 4와 같다.

응급실 간호사의 직무만족에 영향을 미치는 요인의 회귀 모형은 유의한 것으로 나타났다(F = 86.99, *p* < .001). 직무만족에 영향을 미치는 요인은 전문직 자아개념(β = .66, *p* < .001), 팀워크(β = .17, *p* = .003), 응급실 근무 만족(β = .17, *p* = .003)으로 나타났고, 모형의 전체 설명력은 67.7%였다. 즉, 응급실 간호사의 전문직 자아개념이 증가할수록, 팀워크 인식이 긍정적일수록, 응급실 근무 만족도가 만족일수록 직무만족이 높은 것으로 나타났다.

## 논의

본 연구는 응급실 간호사의 전문직 자아개념과 팀워크, 직무만족 정도를 파악하고, 직무만족에 영향을 미치는 요인을 파악하였다.

본 연구에서 응급실 간호사의 직무만족은 평점 3.46점(5점 만점)으로 나타났다. 임상 간호사를 대상으로 시행한 연구에서 평점 3.29점[18], 아동 병동 간호사를 대상으로 시행한 연구에서 평점 3.63점[26]으로 나타났다. 본 연구와 동일한 도구는 아니지만, 점수 범위에서 타부서 간호사의 직무만족과 비교했을 때 응급실 간호사의 직무만족은 중간 정도의 수준이다. 간호조직은 간호단위 별로 업무의 절차와 우선순위가 달라 그 부서 내의 간호사 개인이 인식하는 업무환경 및 내용의 차이가 있고[27] 지역적 특성, 의료기관의 차이, 의료기관별 인력 배치의 차이에서 오는 결과로 부서나 기관에 따라 직무만족 정도는 차이가 나타날 수 있다[28]. 하지만 응급실 간호사의 직무만족이 높으면 간호 업무를 더 잘 수행하고, 개인의 삶의 질 향상에도 영향[5]을 미치기 때문에 응급실 간호사의 직무만족을 지속적으로 살피는 것은 의미가 있다. 직무만족의 하위영역별로 살펴보면, 전문직 간호사로서 책무완수와 전문직 역량 발휘가 가장 높았고, 조직적 차원의 지원과 인정은 가장 낮았다. 임상 간호사 대상 연구에서는 직업의 안정성과 보람 영역이 가장 높았고[18], 아동 병동 간호사 대상 연구에서는 전문직 역량 발휘가 가장 높았다[26]. 전문직 간호사로서의 간호 역량은 단순히 실무능력 뿐만 아니라 가치관, 비판적 사고력, 지식, 마음가짐 등 내재적 요인을 포함하는 것으로, 경험과 교육의 정도에 따라 역량의 강도, 수준이 높아질 수 있다[29]. 따라서 전문지식과 판단력을 기초로 하여 응급환자를 분류하고 우선순위를 신속하게 결정하여 간호를 제공하여야 하는 응급실 특성으로 인해[30] 전문직 간호사로서 책무완수와 전문직 역량 발휘가 높게 나타난 것으로 사료된다. 조직적 차원의 지원과 인정이 낮다는 것은 조직에서 중요한 역할을 하고 있다는 느낌에 대한 만족이 낮은 것으로[25], 선행연구에서도 하위영역 중 가장 낮았다[18,26]. 이는 부서의 차이와 상관없이 간호사의 조직 내 인정과 자부심에 대한 만족도가 낮은 것으로 사료된다.

본 연구에서 응급실 간호사의 전문직 자아개념은 평점 2.63점(4점 만점)으로 종합병원 응급실 간호사[31,32]를 대상으로 시행한 연구와 비슷하였다. 반면, 호주 임상 간호사[20]와 미국 중환자실 간호사[33]의 전문직 자아개념은 평균 3.41점 정도로 상당히 높다. 국외의 경우 간호사에게 지속적인 임상 간호학, 약리학, 병태생리학 등의 교육을 임상에 적용하는 프로그램을 통해 간호사 스스로 발전된다고 느껴[33], 전문직 자아개념이 높게 나타난 것으로 보인다. 전문직 자아개념의 하위영역별로 살펴보면 전문적 실무가 가장 높았고, 의사소통과 만족감이 낮게 나타났다. 전문직 자아개념이 높은 간호사는 자기에 대한 자아개념, 자존감, 자신감, 자부심이 향상되어 간호 전문직의 발전에 기여하므로, 높은 전문직 자아개념은 업무 효율성은 물론 직무만족 증가에도 중요하다[28]. 한편, 응급실 간호사의 팀워크는 평점 3.45점(5점 만점)으로 나타나, 종합병원 임상간호사 대상 선행연구(3.78점)보다 다소 낮았다[17]. 팀워크의 하위영역 중 목표공유가 가장 높았고 상호협력이 가장 낮았다. 본 연구와 동일한 도구는 아니지만, 국외 연구에서도 응급실 간호사의 팀워크가 타부서 간호사보다 비교적 낮았다[34,35]. 대면 대화, 의료기록 등을 포함한 효과적인 의사소통은 팀워크와 질 높은 환자 치료에 필수적인데, 과밀화된 응급실 환경으로 인해 치료 전달 과정이 단편화되어[36] 응급실 간호사의 팀워크 정도가 타부서 간호사보다 낮은 것으로 생각된다.

본 연구에서 응급실 간호사의 직무만족에 영향을 미치는 요인은 전문직 자아개념, 팀워크, 응급실 근무 만족이었다. 그 중 전문직 자아개념이 직무만족에 가장 큰 영향력을 미치는 것으로 나타나, 응급실 간호사가 자신의 전문직 자아개념을 긍정적으로 확고하게 정립할 경우 자신의 업무를 능률적으로 수행할 수 있어 직무만족이 증가한다는 Sung과 Oh[11]의 연구와 같은 맥락이다. 즉, 응급실 간호사의 전문직 자아개념이 긍정적이면 자신의 역할과 책임을 명확하게 인식하고, 직업에 대한 태도 또한 긍정적으로 되어 직무만족이 높아지는 것으로 보여진다. 국외 간호사[20,33]에 비해 본 연구를 비롯한 국내 간호사의 전문직 자아개념이 낮았고 하위 영역 중에서도 의사소통 영역이 낮은 점수였다는 점은 직무만족을 높이는 중요한 선행 전략으로써 전문직 자아개념을 높이는 것이 필수적임을 나타낸다. 이를 위해서 응급실 간호사를 대상으로 약리학, 병태생리학, 임상 추론 등의 지속적인 교육과 효과적인 의사소통을 위한 프로그램의 적용이 필요하다. 지속적인 교육은 지식을 임상에 적용하는 능력 향상으로 스스로 발전한다고 느껴 전문직 자아개념을 높이므로[37], 응급실 간호사의 다양한 교육요구도에 따른 교육 프로그램의 개발 및 적용, 다양한 학술 활동 독려, 대학원 진학 등을 통해 자기 발전이 이루어질 수 있는 기회와 지원이 필요하다.

또한, 응급실 간호사의 높은 팀워크는 의사소통을 통한 임상적 오류를 감소시켜 환자 안전과 만족을 높일 수 있고 응급실 간호사의 직무만족 또한 향상시킬 수 있기에[16,36], 응급실이라는 근무환경적 특성을 팀워크 향상으로 풀어갈 수 있는 단초가 될 수 있다. 실제로 미국의 간호사를 대상으로 의사소통 향상 교육과 매주 아침 담당 의료진의 팀별 논의 시간을 운영한 후 팀워크와 직무만족을 확인하였을 때, 팀워크가 향상되고 환자 치료에 대한 목표와 계획을 공유함으로써 더 나은 환자 간호를 제공할 수 있다는 전문적 자신감이 높아져 직무만족이 높아졌고 이직률이 감소하였다[37]. Rowan 등[38]의 연구에서도 환자 치료를 위해 계획과 개선 방향에 대해 의료진 간 5분~15분 정도 대면으로 의사소통하는 시간을 가졌는데, 이는 팀워크를 강화하고 환자 안전에도 긍정적인 영향을 미쳐 환자만족감 및 직무만족이 증가하였다. 높은 팀워크를 발휘하며 업무에 임할 때 간호사는 자신이 하는 일이 힘들거나 어렵지 않고, 일 하는 자체를 즐겁게 느끼며, 전문직 자아개념 확립을 통해 함께 하고 있는 업무에 대해 더 큰 의미와 가치를 부여하게 되면 함께하는 일의 결과에 만족할 수 있다[17]. 특히, 근무부서 만족 정도는 팀워크의 효과적인 향상과도 일맥상통하므로, 현 근무부서에 만족하는 간호사를 중심으로 팀워크 개선 전략을 수립하는 것이 도움이 될 수 있다.

본 연구를 통하여 응급실 간호사의 직무만족을 높이기 위해 긍정적인 전문직 자아개념 확립과 팀워크 향상의 중요함이 드러났다. 따라서 응급실 간호사의 전문직 자아개념과 팀워크 향상을 통해 직무만족을 높이기 위한 전략으로 응급실에서 자주 겪는 다빈도 질환이나 업무, 상황에 대한 환자 치료 및 간호 이론 교육과, 환자 치료 방향과 관리 전략에 대해 효과적으로 의사소통하는 역량 향상을 위한 팀 시뮬레이션 프로그램의 개발 및 적용을 제언한다.

## 결론

응급실 간호사의 직무만족에 미치는 영향을 확인하기 위한 횡단적 조사연구로, 전문직 자아개념과 팀워크 향상은 응급실 간호사의 직무만족을 높일 수 있었다. 따라서 응급실 간호사의 직무만족을 높이기 위한 전략으로 기초간호 교육과 응급상황 관리 등 다양한 교육요구도에 맞춘 지속적인 교육으로 전문직 자아개념을 높이고 다양한 학술 활동 독려, 대학원 진학 등과 같은 자기 발전이 이루어질 수 있는 기회와 지원이 필요하다. 또한 국내 응급실 환경에 맞는 환자의 치료 계획이나 개선 방법을 공유하며 논의하는 팀 브리핑과 같은 프로그램을 꾸준히 진행하여 의사소통을 증진하고 팀워크를 향상하면 응급실 간호사의 직무만족을 높일 수 있다. 다만, 타 부서와 다른 응급실의 근무환경적 특성을 직접적으로 반영하지 못하였기에 응급실 간호근무환경 등 부서의 환경적 특성을 반영할 수 있는 후속 연구가 필요하다.

## Figures and Tables

**Table 1.** Differences in Job Satisfaction According to the General Characteristics of Emergency Nurses (N = 124)

Characteristics	n (%) or M ± SD	Job satisfaction		
		M ± SD	t / F	*p*
Sex				
Men	16 (12.9)	3.22 ± 0.57	‒1.74	.084
Women	108 (87.1)	3.48 ± 0.59		
Age (yr)	29.79 ± 5.01			
20 ~ 25	19 (15.3)	3.47 ± 0.70	0.89	.448
26 ~ 30	58 (46.8)	3.44 ± 0.56		
31 ~ 35	37 (29.8)	3.41 ± 0.56		
≥ 36	10 (8.1)	3.75 ± 0.67		
Education level				
Diploma (3 years)	14 (11.3)	3.29 ± 0.60	0.89	.414
Bachelor’s (4 years)	104 (83.9)	3.47 ± 0.60		
≥ Master’s	6 (4.8)	3.64 ± 0.52		
Marital status				
Married	29 (23.4)	3.41 ± 0.59	0.58	.565
Single	95 (76.6)	3.48 ± 0.59		
Type of emergency department				
Regional emergency medical center	76 (61.3)	3.50 ± 0.65	1.13	.259
Non-regional emergency medical center	48 (38.7)	3.39 ± 0.48		
Current position				
Charge nurse	29 (23.4)	3.47 ± 0.67	1.30	.259
Staff nurse	95 (76.6)	3.46 ± 0.57		
Career (yr)	6.24 ± 4.50			
< 3	27 (21.8)	3.49 ± 0.63	‒0.08	.933
3 ~ <5	23 (18.5)	3.34 ± 0.40		
5 ~ <10	45 (36.3)	3.47 ± 0.62	0.47	.705
≥ 10	29 (23.4)	3.52 ± 0.65		
Career in emergency department (yr)	4.95 ± 3.68			
< 3	40 (32.3)	3.39 ± 0.59		
3 ~ <5	24 (19.3)	3.38 ± 0.46	1.11	.347
5 ~ <10	44 (35.5)	3.49 ± 0.63		
≥ 10	16 (12.9)	3.69 ± 0.65		
Current work satisfaction				
Satisfied	78 (62.9)	3.67 ± 0.53	5.62	< .001
Dissatisfied	46 (37.1)	3.11 ± 0.53		

M = Mean; SD = Standard deviation.

**Table 2.** Levels of Professional Self-concept, Teamwork, and Job Satisfaction (N = 124)

Variables (range)	M ± SD	Minimum	Maximum
Professional self-concept (1~4)	2.63 ± 0.33		
Practice	2.90 ± 0.38	2.06	4.00
Satisfaction	2.22 ± 0.60	1.00	3.43
Communication	2.25 ± 0.41	1.50	4.00
Teamwork (1~5)	3.45 ± 0.70		
Shared goal	3.48 ± 0.80	1.20	5.00
Outcome-oriented	3.46 ± 0.73	1.50	5.00
Collaboration	3.43 ± 0.75	1.36	5.00
Job satisfaction (1~5)	3.46 ± 0.59		
Recognition from the organization and professional achievement	3.16 ± 0.78	1.22	5.00
Personal maturation through the nursing profession	3.37 ± 0.88	1.33	5.00
Interpersonal interaction with respect and recognition	3.31 ± 0.61	1.75	5.00
Accomplishment of accountability as a nurse	3.98 ± 0.58	2.50	5.00
Stability and job worth	3.74 ± 0.59	2.00	5.00
Display of professional competency	3.98 ± 0.69	2.00	5.00

M = Mean; SD = Standard deviation.

**Table 3.** Correlations among Professional Self-concept, Teamwork, and Job Satisfaction (N = 124)

Variables	Professional self-concept	Teamwork
	r (*p*)	
Teamwork	.44 (< .001)	1
Job satisfaction	.80 (< .001)	.50 (< .001)

**Table 4.** Factors Influencing Job Satisfaction (N = 124)

Variables	B	SE	β	t	*p*	VIF
(Constant)	‒0.31	0.26		‒1.19	.237	
Current work satisfaction (satisfied)^†^	0.21	0.07	.17	3.06	.003	1.17
Professional self-concept	1.19	0.11	.66	10.90	< .001	1.38
Teamwork	0.15	0.05	.17	3.05	.003	1.25
R^2^=.69 Adj R^2^= .68 F(*p*) = 86.99 (< .001) Durbin-Watson = 1.96						

SE = Standard error; VIF = Variance inflation factor. ^†^Reference = dissatisfied.

## Data Availability

Please contact the corresponding author for data availability.

## References

[1] Oh MO, Sung MH, Kim YW (2011). Job stress, fatigue, job satisfaction and commitment to organization in emergency department nurses. Journal of Korean Clinical Nursing Research.

[2] Ju YS (2024 Oct). Emergency medical service statistical research. Final report.

[3] Yang HJ, Lee JH (2021). The effect of the working environment of nurses working in emergency departments in medically vulnerable areas on work dissatisfaction and turnover intention. Health Policy and Management.

[4] Staempfil S, Lamarche K, Perry B (2021). Emergency nursing job satisfaction: challenges and solutions. Nursing Management.

[5] Jang EH, Shim MS (2018). Factors affecting job satisfaction, emotional labor and health promotion behavior of emergency room nurses. Journal of Korean Public Health Nursing.

[6] Motlagh F, Nobahar M, Raiesdang N (2020). The relationship of moral intelligence and social capital with job satisfaction among nurses working in the emergency department. International Emergency Nursing.

[7] Park JA, Yeo JH (2015). Effect of professional autonomy and professional self-concept on job satisfaction of emergency nurses. Journal of Korean Critical Care Nursing.

[8] Lee SY, Lee JS, Kim SY, Lee JY (2017). The effects of job stress and job satisfaction on professional self-concept in nurses. Journal of Digital Convergence.

[9] Choi J, Park HJ (2009). Professional self-concept, self-efficacy and job satisfaction of clinical nurse in schoolwork. Journal of Korean Academy of Nursing Administration.

[10] Cowin L, Johnson M, Craven R, Marsh H (2008). Causal modeling of self-concept, job satisfaction, and retention of nurses. International Journal of Nursing Studies.

[11] Sung MH, Oh MO (2011). The relationships of professional self-concept, role conflict and job satisfaction on emergency department nurses. Journal of Korean Academy of Fundamentals of Nursing.

[12] Goldenhar L, Brady P, Sutcliffe K, Muething S (2013). Huddling for high reliability and situation awareness. BMJ Quality & Safety.

[13] Monroe C, Loresto F, Deutsch S, Kleiner C, Eron K, Varney R (2021). The value of intentional self-care practices: the effects of mindfulness on improving job satisfaction, teamwork, and workplace environments. Archives of Psychiatric Nursing.

[14] Akyuz F, Tengilimoglu D, Ozkanan A, Akyuz S (2022). An examination of the relationships between nurses’ teamwork attitudes, conflicts with college and job satisfaction: an example of public hospital. Hospital Topics.

[15] Baik D, Zierler B (2019). Clinical nurses’ experiences and perceptions after the implementation of an interprofessional team intervention: a qualitative study. Journal of Clinical Nursing.

[16] Grover E, Porter J, Morphet J (2017). An exploration of emergency nurses’ perceptions, attitudes and experience of teamwork in the emergency department. Australasian Emergency Nursing Journal.

[17] Kang SY, Kwon HK, Cho MR (2014). Effects of nurses’ teamwork on job satisfaction at hospital: mediating effect of self-efficacy. The Journal of the Korea Contents Association.

[18] Lee JH, Jung HJ (2022). The influence of professional self-concept, nursing work environment, and teamwork on job satisfaction of nurses in comprehensive nursing care ward. The Journal of the Convergence on Culture Technology.

[19] Faul F, Erdfelder E, Buchner A, Lang A (2009). Statistical power analyses using G*Power 3.1: tests for correlation and regression analyses. Behavior Research Methods.

[20] Arthur D (1995). Measurement of the professional self-concept of nurses: developing a measurement instrument. Nurse Education Today.

[21] Sohng KY, Noh CH (1996). An analytical study of the professional self-concept of hospital nurses in Korea. Journal of Korean Academy of Nursing.

[22] Larson C, Lafasto F (1989). Teamwork: what must go right / what can go wrong.

[23] Kim TB (2006). Influence of teamwork and leadership on organizational effectiveness of hospital employees [dissertation].

[24] Cho MR (2012). Relationship of nurses’ teamwork to organizational effectiveness at hospital [master’s thesis].

[25] Lee BS, Eo YS, Lee MA (2018). Development of job satisfaction scale for clinical nurses. Journal of Korean Academy of Nursing.

[26] Kim HE, Park JH, Seo EJ, You MA (2022). The effects of emotional labor and grit on job satisfaction among pediatric nurses. Journal of Korean Clinical Nursing Research.

[27] Won HJ (2018). Impacts of psychosocial work environment on nurses’ job satisfaction based on the type of hospital departments. The Korean Journal of Health Service Management.

[28] Kim JM, Lee YM (2019). Factors influencing job satisfaction among nurses in integrated nursing care service wards. The Korean Journal of Health Service Management.

[29] Chang YH, Cho YS, Kwak MJ (2006). A study of factors related nursing competency in nurses. Clinical Nursing Research.

[30] Kim JH, Ahn HY, Eom MR, Lee MY (2010). A study of emergency room nurses’ burn out, nursing performance, and professional identity. Korean Journal of Occupational Health Nursing.

[31] Sung MH, Choi EY (2012). The relationships between professional self-concept, nursing performance and retention intention of emergency department nurses. Journal of Korean Academy of Fundamentals of Nursing.

[32] Kim MR, Seo EJ, Shin SH (2019). The influence of the emotional labor, professional self-concept, self-efficacy & social support of emergency room nurse’s burnout. Korean Journal of Stress Research.

[33] Kelly S, Courts N (2007). The professional self-concept of new graduate nurses. Nurse Education in Practice.

[34] Kalisch B, Lee HH (2009). Nursing teamwork, staff characteristics, work schedules, and staffing. Health Care Management Review.

[35] Kalisch B, Lee KH (2013). Variations of nursing teamwork by hospital, patient unit, and staff characteristics. Applied Nursing Research.

[36] Kilner E, Sheppard L (2010). The role of teamwork and communication in the emergency department: a systematic review. International Emergency Nursing.

[37] Baik D, Zierler B (2018). RN job satisfaction and retention after an interprofessional team intervention. Western Journal of Nursing Research.

[38] Rowan B, Anjara S, Brun A, Macdonald S, Kearns E, Marnane M (2022). The impact of huddles on a multidisciplinary healthcare teams’ work engagement, teamwork and job satisfaction: a systematic review. Journal of Evaluation in Clinical Practice.

